# Systematic Review on Internet Support Groups (ISGs) and Depression (1): Do ISGs Reduce Depressive Symptoms?

**DOI:** 10.2196/jmir.1270

**Published:** 2009-09-30

**Authors:** Kathleen M Griffiths, Alison L Calear, Michelle Banfield

**Affiliations:** ^1^Centre for Mental Health ResearchThe Australian National UniversityCanberraAustralia

**Keywords:** Depression, consumer participation, Internet, self-help groups

## Abstract

**Background:**

Internet support groups (ISGs) enable individuals with specific health problems to readily communicate online. Peer support has been postulated to improve mental health, including depression, through the provision of social support. Given the growing role of ISGs for both users with depression and those with a physical disorder, there is a need to evaluate the evidence concerning the efficacy of ISGs in reducing depressive symptoms.

**Objective:**

The objective was to systematically review the available evidence concerning the effect of ISGs on depressive symptoms.

**Method:**

Three databases (PubMed, PsycINFO, Cochrane) were searched using over 150 search terms extracted from relevant papers, abstracts, and a thesaurus. Papers were included if they (1) employed an online peer-to-peer support group, (2) incorporated a depression outcome, and (3) reported quantitative data. Studies included both stand-alone ISGs and those used in the context of a complex multi-component intervention. All trials were coded for quality.

**Results:**

Thirty-one papers (involving 28 trials) satisfied the inclusion criteria from an initial pool of 12,692 abstracts. Sixteen trials used either a single-component intervention, a design in which non-ISG components were controlled, or a cross-sectional analysis, of which 10 (62.5%) reported a positive effect of the ISG on depressive symptoms. However, only two (20%) of these studies employed a control group. Only two studies investigated the efficacy of a depression ISG and neither employed a control group. Studies with lower design quality tended to be associated with more positive outcomes (*P* = .07). Overall, studies of breast cancer ISGs were more likely to report a reduction in depressive symptoms than studies of other ISG types (Fisher *P* = .02), but it is possible that this finding was due to confounding design factors rather than the nature of the ISG.

**Conclusions:**

There is a paucity of high-quality evidence concerning the efficacy or effectiveness of ISGs for depression. There is an urgent need to conduct high-quality randomized controlled trials of the efficacy of depression ISGs to inform the practice of consumers, practitioners, policy makers, and other relevant users and providers of online support groups.

## Introduction

Internet support groups (ISGs) provide individuals with specific health problems an opportunity to share experiences and to seek, receive, and provide information, advice, and emotional support online. It has been estimated that millions of people visit online peer-to-peer discussion groups daily [1], and there is evidence that over 28% of Internet users have visited an online support group at least once [2].

Internet users seeking health information frequently access information about depression [3], and online depression groups have been reported to be among the most common ISGs on the Internet [4]. It is also known that there is a high level of depression among individuals with a physical illness [5]. Thus, many users seeking to join health ISGs may have elevated depressive symptoms or may be at risk of developing depression.

Peer support has been postulated to improve mental health, including depression, through the provision of social support, which alters cognitions, attitudes, self-attributions, and coping, which, in turn, leads to a reduction in depressive symptoms [6]. Given the growing role of ISGs for both consumers with depression and other health conditions, there is a need to evaluate the evidence concerning the effect of these groups on depressive symptoms. One research group has conducted a high-quality, systematic review of studies on the effect of health ISGs on a range of outcomes [1]. The review did not, however, focus on depression outcomes in detail and was confined to articles published prior to October 2003.

The current paper aims to provide a systematic and comprehensive review of the available evidence concerning the effect of ISGs on depressive symptoms regardless of the ISG health condition. A more detailed review of depression ISGs specifically is provided in a companion paper, which reports the scope and findings from all qualitative and quantitative empirical studies of depression ISGs (see [7]).

## Methods

### Databases

Three databases (PubMed, PsycINFO, Cochrane) were searched using keywords and phrases for the period prior to August 2007. The search was undertaken at two time points, the first in May 2005 and the second in July 2007.

### Search Methodology

The search terms and strategies were based on those reported by Eysenbach et al [1], which involve the following concepts: (computer/Internet communication *and* support) *or* e-community venue. In addition, a further 48 relevant search terms were extracted from research papers on ISGs, abstracts extracted by running database searches using the resulting search terms, and an online thesaurus searching for similes of key terms [8].

### Study Identification

**Figure 1:**
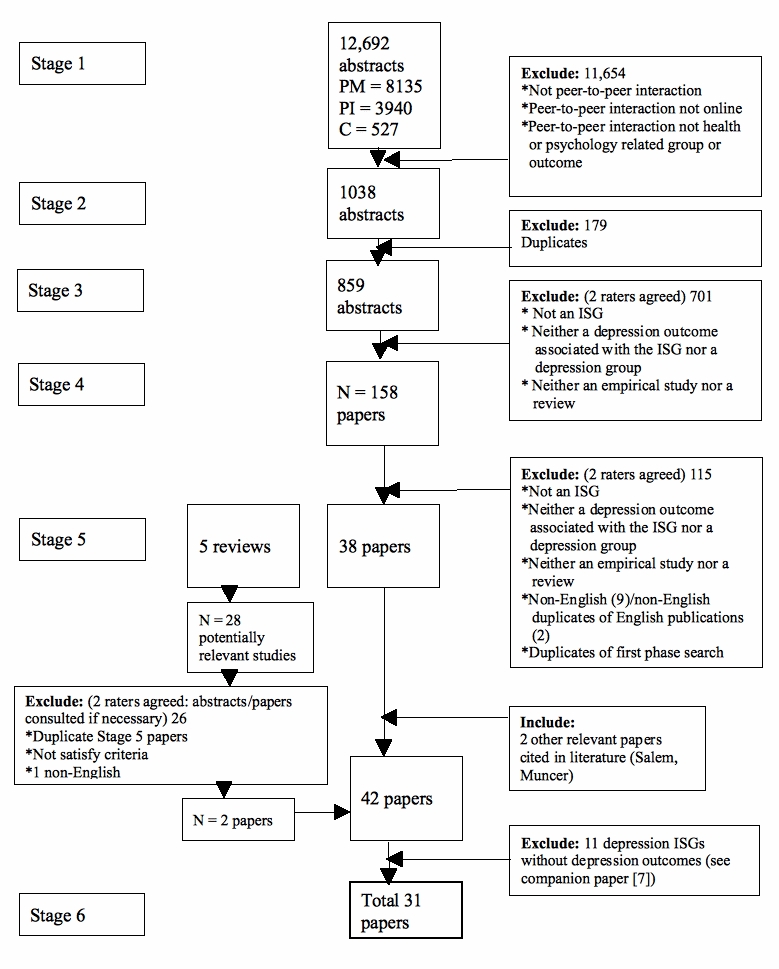
Study identification flow diagram: PubMed (PM), PsycINFO (PI), Cochrane (C)

A multi-step process was employed to select relevant studies for the current review and the review of depression ISGs reported in the companion paper to this study [7] (see Figure 1). In the first stage, each of the 12,692 abstracts returned by the database searches was screened by one of the three authors (AC, MB, KG). The aim of this stage was to screen out clearly irrelevant abstracts and, in particular, to eliminate any reference that clearly did not satisfy the following inclusion criteria:

1. Study discussed or investigated peer-to-peer interaction.
2. Study discussed or investigated at least one of the following: online/electronic support groups, online/electronic social or peer support, online/computer-based communication or interaction, collaborative virtual environments or interventions.
3. The support “group” discussed or investigated was health/psychology related (eg, biological illness, mental illness, health risk factors, bereavement, group counseling), or the article measured a health/psychology related outcome in relation to the support group.

After removing duplicate papers (Stage 2), the remaining abstracts (n = 859) were coded as relevant, not relevant, or possibly relevant according to the following inclusion criteria:

1. Employed an online peer-to-peer support group
2. Incorporated either a depression outcome or involved a unipolar depression ISG
3. Reported either quantitative or qualitative empirical data (Stage 3)

Studies were included whether they incorporated a stand-alone ISG or involved a complex multi-component intervention. Reviews of ISGs satisfying the first two criteria were identified and analyzed separately. Abstracts were coded by one author (AC or KG) and checked by a second author (KG or AC). Any disagreement was resolved by discussion. After excluding the irrelevant abstracts, 158 papers were obtained, read (if in English), and coded against the inclusion criteria by one author (KG). The coding was checked by a second author (AC). Those papers that did not report a depression outcome or did not concern an ISG exclusively devoted to depression were excluded (Stage 4), as were any duplicate papers generated as a result of conducting a two-phase searches process (n = 2). In addition, two papers were judged to be non-English versions of an English-language publication and were excluded [9,10]. Nine other non-English papers of possible but not definite relevance were excluded for pragmatic reasons (cost of translation) [11-19]. It is unclear how many of these would have been retained in the review had they been formally translated. However, one did not satisfy the inclusion criteria based on a translation by the first author [18], and only three of the remaining non-English papers were rated as probable or definite relevance based on the English abstract and a perusal of the content of the tables in the untranslated paper [11,14] or a partial translation supplied by a colleague [19].

The above process yielded a total of 38 relevant papers and five systematic reviews. Two additional relevant papers were identified from the five reviews, and a further two papers cited in at least one of the 38 relevant papers were included among the pool of relevant papers (Stage 5). This resulted in a total of 42 relevant papers of which 31 papers comprising 28 separate trials incorporated a depression outcome (Stage 6) and 11 (studies of depression ISGs) did not. The current paper focuses on the 28 trials reporting a depression outcome.

### Coding of the Included Papers

The 31 papers reporting a depression outcome were independently coded by two raters (KG, AC), and discrepancies were subsequently resolved by discussion between the two raters.

Quantitative studies that included depression outcomes were coded for ISG, participant and study characteristics, and depression outcomes. The ISG characteristics extracted included the psychological or physical condition experienced by members of the group, the format of the ISG (newsgroup, bulletin board, chatroom), whether moderated (yes, no, don’t know), and, if so, by whom (consumer, health professional, both, don’t know), ISG type (public, research, other restricted access), and ISG origin (United States, Europe, other). Participant characteristics recorded included age (median older than 25 years or 25 years and younger), gender, education, ethnicity, and rurality. Study design characteristics and quality were also coded, including sample size, attrition, design type (randomized controlled trial [RCT], controlled trial, historical control, pre-post, cross-sectional, case series), appropriateness of randomization process and reporting, whether the study employed an intent-to-treat (ITT) analysis (yes, no), and how missing data were treated (last observation carried forward, multiple imputation, other). Each study was also rated as to whether it involved a multi-component design of which the ISG was just one component, or whether the study evaluated a stand-alone ISG or at minimum used a control group that controlled for the non-ISG components of the intervention. Intervention characteristics recorded included duration of intervention and length of longest follow-up. The depression outcome measures used in each study were recorded, and each sample was rated according to whether it yielded a statistically significant positive outcome. Finally, raters coded the type of publication (thesis, journal, book), country of primary author (United States, Europe, other), and whether consumers were actively involved in the design or conduct of the research.

### Analyses

A formal quantitative meta-analysis was not conducted due to the low quality of the studies meeting the inclusion criteria and the heterogeneous nature of the conditions studied. However, the possible role of different characteristics and quality were explored by comparing the characteristics of samples reported to have yielded positive, statistically significant results with those that did not, using a series of Fisher exact tests for categorical attributes and Mann-Whitney tests for other data. For the purposes of this analysis, data were analyzed at the comparison rather than the study level. In addition, for descriptive purposes, where possible, Cohen’s d standardized effect sizes were calculated and reported. For uncontrolled studies, the pre-post standardized effect size was calculated from the mean pre-test and post-test scores and standard deviations. For controlled studies, the study effect size was the difference between the pre-post effect size for the control group and the pre-post effect size for the intervention group. In a study involving the comparison between depression scores for high-use compared to low-use Internet users, effect size was based on the standardized difference for the two groups. Effect sizes were not calculated in several instances. Where only the *t* test value for dependent (or equivalent) samples was available [20], no effect size was estimated as such *t* values are based on the standard error of the difference rather than a pooled standard deviation and therefore overestimate the effect size. For the same reason, an effect size was not calculated from the F value of simple effects analysis of residualized change in depression [21]. In addition, effect sizes were not calculated for studies in which only baseline adjusted means [22] and baseline adjusted difference in change [23] were reported and for one study containing apparent inconsistencies in reported sample standard deviations [24].

## Results

Of the 28 studies with depression outcomes, five reported results separately for two different populations (patient versus carer [21,25], mothers versus fathers [26], adolescents versus young adults [27], heterogeneous versus homogenous group composition [28]), and one involved two arms differing in intervention duration [24]. Thus, there were a total of 34 samples. In reporting the findings below, the term “samples” will be used to refer to these 34 different populations or arms, and the term “studies” will be reserved to describe the 28 trials.

### Study Characteristics

Of the 28 studies with depression outcomes, 16 involved the evaluation of stand-alone ISGs or used a design that controlled for the use of intervention components other than the peer-to-peer component or involved cross-sectional studies of online groups (single component). The remaining studies incorporated a multi-component intervention that comprised the discussion group plus at least one additional component such as health education, skills training, or decision aids. Table 1 and Table 2 present the characteristics of each of the single-component and multi-component studies with a depression outcome. Table 3 summarizes the intervention and design characteristics across studies (ISG format and type, level of evidence) or, where appropriate, across samples (conditions, participant characteristics). Complete data were not available for all variables.

**Table 1:** Study characteristics and findings for single-component or cross-sectional studies^a^

Study	Participants	Design/Control	Intervention/Nature of ISG	Outcome Measures/ Follow-Up	ITT	Completer No. and % Dropout (d/o)	Results/Effect Size^b^	Significant?
**BreastCancer**								
Winzelberg 2003 [29] USA	N = 72 women with BC, diagnosed in past 32 mths I = 36; C =36 Recruitment: Advertisements in media and oncology offices	RCT/WLC Randomization method not specified	12-wk Web-based structured newsgroup ISG (Bosom Buddies) One topic/week introduced by psychologist moderator (3 consecutive groups: n = 10, n = 11, n = 15)	CES-D Baseline 12 wks	Yes LOCF	N = 58 (19.4% d/o) I = 28 (22.2% d/o) C = 30 (16.6% d/o) Baseline measures did not predict dropout	Greater reduction in depressive symptoms in ISG group than control ES = 0.60 (completers)	Yes
Lieberman 2003 [30] USA	N = 32 women with BC Recruitment: Online advertisement on BC websites and via media, physicians, hospitals, and community centers	Pre-post	16 week × 1.5 hr chatroom sessions with experienced leader therapist plus 24 hr/day bulletin board access	CES-D Baseline 16-20 wks	No	I = 26 (18.8% d/o) Predictors of non-adherence: poorer coping with anxiety, more fatalistic, pain interfered less with life, less perceived change in relationships/personal strength	Significant reduction in depressive symptoms after use of ISG ES = 1.05	Yes
Lieberman 2005 [31] USA	N = 114 women with BC who joined 1 of 5 frequently used public bulletin boards < 8 wks previously Recruitment: Advertisement on the online bulletin board	Pre-post	6- to 8-mth membership on public BC moderated bulletin board ISG providing emotional support	CES-D “Baseline” 6 mths post baseline	No	6 mths I = 91 (20% d/o) NS difference between completers and non-completers demographics, clinical characteristics, depression severity, posttraumatic growth/psychosocial well-being	Significant reduction in depressive symptoms after use of ISG ES = 4.52	Yes
Lieberman 2006 [20] USA	N = 74 women with BC who joined 1 of 4 frequently used bulletin boards < 8 wks previously Recruitment: Advertisement on the online bulletin board	Pre-post	6- to 8-mth membership on public BC bulletin board providing emotional support No information about moderator status	CES-D “Baseline” 6 mths post baseline	No	6 mths I = 61 (17.6% d/o) Baseline depression severity did not predict dropout	Significant reduction in depressive symptoms after use of ISG	Yes
Rodgers 2005 [32] USA	N = 100 randomly selected women with BC who posted to a BC bulletin board during particular 1-wk period	Pre-post	Variable duration membership (mean 247 days; range 44-1001 days) of public BC bulletin board	Thematic analysis of mood		I = 100 (only followed up while members)	Significant association between frequency of posting and improved mood 43.3% participants improved mood (no data on poorer mood)	Possibly
**Mental Disorder**								
Andersson 2005 [33] Sweden	N = 60 participants with depression (CIDI diagnosis major depression and MADRS-S score 15-30 [mild to moderate depression]) Recruitment: Press release/media	Pre-post arm^c^	10-wk moderated bulletin board ISG	BDI MADRS-S (completer analysis only) Baseline 10 wks 36 wks	Yes LOCF	Post-treatment I = 35 (41.7% d/o) NS between completers and non-completers in baseline depression, quality of life, treatment history, demographic characteristics	NS reduction in depressive symptoms with use of ISG MADRS-S: ES = 0.34 (10 wks) ES = 0.87 (36 wks) (ES values not ITT)	No (ITT and completers)
Houston 2002 [34] USA	N = 103 users of public depression ISGs N = 89, 86.4% depressed on CES-D Recruitment: Requests for volunteers on listservs/bulletin boards	Pre-post	Participation in public listservs/bulletin boards at least 12 mths	CES-D “Depression” = CES-D ≥ 23 at least 1-2 mths after start bulletin board “Baseline” 6 mths 12 mths	No	6 mths I = 72 (30.1% d/o) 12 mths I = 66 (35.9% d/o) Of those depressed at baseline, 79 completed at least 1 follow-up (20.2% d/o) Attrition not predicted by baseline severity of depression, frequency of ISG use, or social support	Resolution of depression greater in more frequent ISG users after adjustment for baseline depression severity/ demographic variables (*P* < .03)	Yes
Golkaramnay 2007 [35] Germany	N = 228 adults discharged from psychiatric hospital with non-psychotic mental disorder I = 114 (with TK insurance - 61 mood disorder) C = 114 (without TK insurance - 59 mood disorder)	CT/TAU	12- to 15-wk exposure to psychotherapist-guided chatroom ISG comprising 8-10 people for 90 mins/wk	LIFE semi- structured interview 1 wk 12 mths	No	I = 97 (14.9% d/o)^d^ C = 104 (8.8% d/o)^d^	NS difference in the percentage of ISG and control participants with a diagnosis of disorder at 12 mths follow-up	No
**Diabetes**								
McKay 2002 [36] USA Glasgow 2003 (12 month f/up) [37] USA	N = 160 primary care DB patients aged 40 to 75 yrs with no Internet access at home or work I_1= 40^e^; C = 40 Recruitment: Letters sent by primary care physicians to their patients with DB	RCT /info control^f^ Randomization method not specified	I_1: 10-mth professionally moderated bulletin board/chatroom and information^g^	CES-D 3mths 10 mths	No	3 mths: N= 133 (16.9% d/o) I_1= 30 (25% d/o) C= 33 (17.5% d/o) 10 mths: 18% d/o overall; further details not provided Characteristics of completers and dropouts did not differ	No effect of ISG on reduction in depressive symptoms at either follow-up period ES = 0.15 (3 mths)	No
**Renal**								
Quick 1999 [38] USA	N = 3 people undergoing dialysis for renal disease Recruitment: Dialysis clinics, dialysis websites	MT (single case)	5-wk participation in a pre-existing public email discussion list ISG for renal patients No information about moderation status	BDI 3 time points	Yes	N = 3 (0% d/o)	No improvement in depressive symptoms over time	No
**NoDisorder**								
Gross 2006 [27] USA	N = 77 adolescents aged 11 to 15 yrs N = 81 first-year college students Recruitment: Adolescents - Summer camps/after-school programs College students ***-*** Fliers/ announcements in college dorms/halls/classrooms; in person recruitment at halls Rewards for participation/ completing consent form	RCT^h^ Randomization method not specified	12 mins of instant online messaging to an unknown peer after experimental induction of low mood in control and intervention group	Dysphoria measure devised for study Baseline and immediate post intervention	No	Adolescents^i^: N = 50 (35.1% d/o, including 1 participant dropped by researchers) College students: N = 60 (25.9% d/o, including 14.8% dropped by researchers)	Adolescents^j^: Mood improvement greater for peer-to-peer intervention group than control College students: No difference in mood change for peer-to-peer group compared to control	A:Yes C:No
Shaw 2002 [39] USA	N = 46^k^ introductory psychology university students Recruitment: Advertisement on a psychology course Web page	MT	4-8 wks of online chat sessions with the same anonymous partner Participant provided with topics for the chat	CES-D Pretest, mid-test, post-intervention	No	I = 40 (13%^k^ d/o)	Significant reduction in depressive symptoms following use of ISG ES = 0.47	Yes
Morgan 2003 [40] USA	N = 287 (or 256) first-year residential university students Recruitment: Postal notification followed by email	XS	Chatroom unspecified /instant messaging	Modified CES-D (11-item, Iowa version)	N/A	N/A	Significant correlation between chatroom hrs and depressive symptoms r = −.13, *P* < .05 Increased chatroom hrs predicted decreased depression after controlling for demographic variables/social support *P* < .01	Yes
Sun 2005 [41] USA	N = 2373 7th grade students (age 11 to 16 yrs) Recruitment: Invitation via school	XS	Chatroom unspecified	Not specified	N/A	N/A	Daily chatroom users more depressed than those with Internet access who did not use chatrooms OR 1.2, *P* < .05	Yes (-ve effect)
Campbell 2006 [42] Australia	N = 188 self-selected global sample of online users of whom 137 were frequent chat users and 51 were not Recruitment: Passive recruitment via website advertisement (eg, on APA website)	XS	High chatroom (unspecified) use Control low chatroom (unspecified) use	ZDS DASS	N/A	N/A	NS difference in depressive symptoms for high chatroom compared to low chatroom use ES = −.06 (ZDS) ES = 0.02 (DASS)	No
Kang 2007 [43] USA	N = 158 chatroom users from US university community (57% female) Recruitment: Not reported	XS	Chatroom unspecified	CES-D Kraut depression items	N/A	N/A	Higher chatroom use predicted lower depression β = −0.29, *P* < 0.001	Yes

^a^ APA = American Psychological Association; BC = breast cancer; BDI = Beck Depression Inventory; C = control sample size; CES-D = Center for Epidemiologic Studies Depression Scale; CIDI = Composite International Diagnostic Interview; CT = controlled trial; DASS = Depression Anxiety Stress Scales; DB = diabetes; ES = effect size; I = intervention sample size; ITT = intent to treat; LIFE = Longitudinal Interval Follow-up Evaluation; LOCF = last observation carried forward; MADRS-S = Montgomery-Asberg Depression Rating Scale; MT = multiple time points; N/A = not applicable; OR = odds ratio; NS = no significant difference; RCT = randomized controlled trial; TAU = treatment as usual; TK = Techniker Krankenkassde; WLC = wait list control; XS = cross-sectional; ZDS = Zung Depression Scale.

^b^ Pre-post standardized effect size (for pre-post design) or difference between intervention and control pre-post effect sizes (for controlled designs).

^c^ This study was an RCT involving an intervention group comprising CBT self-help and an ISG and a control group involving an ISG alone. This design does not permit an evaluation of the effect of ISG alone. Therefore, only the data for the control group (pre-post) are presented here.

^d^ Did not complete both baseline and follow-up assessments; other dropout information not available.

^e^Also, I_2 = 40, I_3 = 40.

^f^ Online articles on diabetes (information only).

^g^Also two other conditions: I_2: access to professional coach and blood glucose tracking; I_3: a combination of I_1 & I_2.

^h^Participants randomized to one of three groups: (1) control, (2) intervention, (3) intervention group partners.

^i^ These figures are for participants across all groups including dyad partners who had not undergone negative mood induction. Sample size and dropout figures were not available for the groups separately.

^j^ Outcome measures recorded and analyzed for mood induction intervention and control samples only.

^k^ Unclear if n = 46 before or after consent.

**Table 2:** Study characteristics for multi-component interventions^a^

Study	Participants	Design/Type of Control	Intervention/Nature of ISG	Outcome Measures/ Follow-Up	ITT	Completer No. and % Dropout (d/o)	Results/Effect Size^b^	Significant?
**Cancer**									
Owen 2003 [22] USA	N = 59 women with BC I = 29; C = 30 Recruitment: Contact with patients in medical oncology clinics, advertisements in hematology/oncology outpatient clinic, health websites, community nurse referral, media $10 for completing each survey	RCT/WLC Randomization: Random number generator	12-wk SURVIVE online program comprising health professional, moderated bulletin board group, cancer information, resources, self-management advice, art/poetry forum, structured coping skills exercises (including stress management, assertiveness, and structured problem solving training) Up to 20 participants per group	HADS Baseline 12 wks	No	I = 25 (13.8% d/o) C = 27 (10% d/o)	NS difference in baseline adjusted mean at 12 wks for intervention and control groups	No
Van Den Brink 2007 [23] Netherlands	N = 184 people post-surgery for head or neck cancer I = 39; C = 145 Recruitment: Tertiary university hospital–treated patients recruited by doctor independent of treating physicians	CT/TAU	6-wk electronic health information support system comprising peer-to-peer forum and email communication; information and monitoring via electronic questionnaire	“Feelings of depression” Baseline 6 wks 3 mths	No	N = 163 (11.4% d/o) I = 35 (10.3% d/o) C = 128 (11.7% d/o)	NS baseline adjusted difference in change at 6 or 3 mths for intervention compared to control groups	No
**Neurological**									
Brennan 1995 [44] USA	N = 102 caregivers of people with Alzheimer’s disease I = 51; C = 51 Recruitment: Research registry, support groups	RCT/TAU Randomization: Not specified	12-mth access to bulletin board moderated by nurse who posted messages to “foster systematic group cohesion” and information and decision support (expert Q&A)	CES-D Baseline 12 mths (intervening variable)	No	I = 47 (7.8%^c^ d/o) C = 49 (3.9% d/o)	Depression was treated as a intervening variable rather than an outcome ES = 0.24	N/R
Liebermann 2005^d^ [28,45] USA	N = 66 or 65 patients with PD assigned to: Heterogeneous (Het) groups - variable age and time since diagnosis Homogenous (Hom) groups - homogenous age and time since diagnosis^d^ Recruitment: Fliers to support groups, PD clinic, practitioners, online posts, newsletter	Pre-post	20 wks × 1.5 hrs weekly health-professional moderated chatroom and bulletin board available at all times and Q&A weekly health education session with an expert	CES-D Baseline 20 weeks	No	Dropout rates could not be calculated separately for Hom and Het Combined: I = 32 (39% d/o)^d^ NS differences in baseline measures between dropouts and completers	Significant reduction in depressive symptoms following intervention involving Hom but not Het ISG^d^	Hom: Yes Het: No
**Chronic Illness**									
Battles [46] USA	N = 32 children (age 8-19 yrs) with serious chronic illness (HIV, cancer, granuloma, neurofibromatosis) participating as residential out patients in pediatric clinical trials at the NIH Recruitment: Playroom staff at NIH residential center identified potentially eligible participants Researchers approached eligible participants/parents	(1) Restricted randomly alternating (A, B) treatment design Control = normal playroom activity (2) Pre-post	4 × 30 min sessions on the STARBRIGHT World (SBW) program comprising network connection to other children in a hospital (video) Connect/Find a Friend and information about medical conditions and entertainment and distraction Sessions administered across multiple NIH residential visits over unspecified time period	Depression Analogue Scale CBCL-anxious depressed (parent) Usefulness in reducing sadness-depression (parent) Pre-post session Pre-post intervention	d/k	d/k	NS improvement in depression ratings or symptoms 24% parents reported positive effects of the program on mood Estimated ES (CBCL) = −0.06	No
Hill 2006 [47] USA	N = 120 female, rural residents (35 to 65 yrs) with chronic illness (diabetes/rheumatoid condition/ heart condition/multiple sclerosis/cancer) I = 61; C = 59 Recruitment: Mass media, agency and service organization newsletter, and word of mouth	RCT^d^ Randomization: Method not specified	22-wk professionally moderated online support group and online health information modules The support group was described as an “asynchronous chatroom”	CES-D Baseline 22 wks	No	I = 43 (29.5% d/o) C = 57 (3.4% d/o)	NS differences in reduction in depressive symptoms in intervention compared to the control group ES = 0.15	No
**Carers**									
Bragadottir 2004 [26] USA thesis, Icelandic sample	N = 21 parents of children who had completed cancer treatment within past 5 yrs Mothers: I = 11 Fathers: I = 10 Recruitment: From files of Icelandic hospital responsible for treating children with cancer	Pre-post	4-mth access to health professional–moderated mailing list Professionals facilitated and joined in group discussions, answered questions, directed parents to resources, corrected misconceptions/misinformation, monitored appropriateness of discussions	SCL-90 depression subscale Baseline 3 mths 4 mths	No	3 mths and 4 mths N= 16 (23.8% d/o) Mothers: I = 8 (27.3% d/o) Fathers: I = 8 (20% d/o)	Mothers: NS reduction in depressive symptoms ES = −0.10 (3 mths) ES = 0.20 (4 mths) Fathers: NS reduction depressive symptoms ES = −0.22 (3 mths) ES = 0.40 (4 mths)	Mothers: No Fathers: No
**Carers andHeart Recipients**									
Dew 2004 [21] USA	N = 124 heart recipients and family caregivers Recipients: I = 24; C = 40 Caregivers: I = 20; C = 40 Recruitment: Letter from transplant team asking if had Internet access Those with access “approached” to participate	Controlled/ “Historic” TAU comparison group enrolled in other longitudinal studies and matched for demographic distribution and assembled before or after intervention	4-mth HeartNet programs comprising discussion groups (online moderated bulletin boards, separate caregiver and recipient boards) and interactive online stress and medical regimen management skills training grounded in CBT principles and Ask an Expert (online questions to transplant team expert plus Q&A Library plus archived responses to Ask and Expert plus Health living tips plus Resources plus References Library)	SCL-90 Depression subscale Baseline, 4 mths (I), and 4-6 mths (C)	No	Recipients: I= 20 (16.7% d/o) C = 34 (15% d/o) Caregivers: I= 17 (15% d/o) C = 34 (15% d/o)	Recipients: Receiving intervention showed a greater reduction in depressive symptoms than the control group Caregivers: NS difference in reduction in depressive symptoms in intervention compared to the control group	Recipients:Yes Caregivers: No
**Diabetes**								
McKay 2001 [48] USA	N = 78 sedentary people with type 2 diabetes aged 40 years or older I = 38; C = 40 Recruitment: Email postings to online diabetes groups and websites	RCT /online information, blood glucose tracking Control Randomization: Automatic system allocated	8-wk D-Net Active Lives program comprising tailored online physical activity program with tracking of daily physical activity, information about a physical activity plus online personal coach counseling plus health professional moderated online peer support (Active Lives Support Group)	CES-D Baseline 8 wks	No	N = 68 (13% d/o) I = 35 (7.9% d/o) C = 33 (17.5% d/o) Predictors of drop out: None	NS difference in reduction in depressive symptoms in intervention compared to control group ES = 0.35	No (*P* = .10)
**HIV**									
Gustafson 1994 [49] USA Gustafson 1999 [24] USA	N = 219; I = 118; C = 97 with HIV 3-mth intervention: I = not specified; C = not specified 6-mth intervention: I = not specified; C = not specified Recruitment: Posters, newspaper advertisement, HIV clinics/organizations Paid to complete surveys	RCT/TAU Randomization: Independent third party using random number table	6 mths (Cohort 1) and 3 mths (Cohorts 2 and 3) CHESS program comprising online facilitated bulletin board discussion group plus Q&A plus Instant Library (information articles) plus Ask an Expert (communication with medical experts) plus Getting help/support plus Referral Directory plus Personal stories plus assessment (of lifestyle risks) plus Decision Aid plus Action Plan for implementing decisions	MOSdepression subscale 3-mth Int: Baseline, 2 mths, 5 mths 6-mth Int: Baseline, 2 mths, 5 mths, 9 mths	No	Dropout rates could not be calculated separately for 3-mth and 6-mth intervention groups All cohorts at 2 mths: I = 97 (17.7% d/o) C = 90 (9.3% d/o) All cohorts who “completed trial”: I = 94 (21% d/o) C = 89 (9.2% d/o)	NS differences in reduction in depressive symptoms in intervention compared to control group for any follow-up/cohort combination	3-mth Int (5-mth f/up): No 6-mth Int (9-mth f/up): No
**MentalDisorder**									
Taylor 2006 [50] USA	N = 480 college women (18 to 30 yrs) at high risk of developing an eating disorder I = 244; C = 236 Recruitment: Flyers at colleges, campus mailings, mass media	RCT/WLC Randomization: Stratified by school; computer-generated sequences produced by study coordinator	8-wk professionally modified bulletin board and cognitive behavioral intervention	CES-D Baseline 8 weeks 60 weeks	No	I = 191 (21.7% d/o)^e^ C = 198 (16.1% d/o)^e^ NS demographic or baseline differences between completers and non-completers	NS difference in reduction in depressive symptoms in intervention compared to control group ES = 0.04 (8 wks) ES = 0.11 (60 wks)	No (*P* < .07)
**IVF**									
Tuil 2006 [25] Netherlands	N = 244 participants undergoing IVF or ICSI treatment in authors’ hospital Males: I = 61; C = 61 Females: I = 61; C = 61 Recruitment: From author IVF clinic	RCT “Randomization”: Alternating allocation to intervention or control	Access to professionally moderated bulletin board and chatroom (for communication with peers and professionals) plus information and access to own records during period of IVF/ICSI treatment cycle	Beck Depression Index for Primary Care Baseline Post-intervention	No	Males: I= 51 (16.4% d/o) C = 38 (37.7% d/o) Females: I= 51 (16.4% d/o) C = 40 (34.4% d/o)	Males: ES = −0.25 Females: ES = 0.18	Males: No Females: No

^a^ BC = breast cancer; C = control sample size; CBCL = Child Behavior Checklist; CBT = cognitive behavioral therapy; CES-D = Center for Epidemiologic Studies Depression Scale; CT = controlled trial; d/k – don’t know; ES = effect size; HADS = Hospital Anxiety & Depression Scale; I = intervention sample size; ITT = intent to treat; MOS = Medical Outcomes Study; NIH = National Institutes of Health; N/R = not reported; PD = Parkinson’s disease; RCT = randomized controlled trial; SCL-90 = Symptom Checklist 90; TAU = treatment as usual; WLC = wait list control.

^b^ Pre-post standardized effect size (for pre-post design) or difference between intervention and control pre-post effect sizes (for controlled designs).

^c^ Includes three (5.9%) dropouts “not able to have computer installed.”

^d^ Due to apparent inconsistencies within and between the two papers on this study, effect sizes have not been computed, individual sample sizes are not reported, and individual dropout rates not computed.

^e^ Computed for completers of CES-D only; data for overall completers not available.

**Table 3:** Study and sample characteristics^a^

Study (Sample^c^) Variable	Total n = 28 (n = 34)^c^	Single Component n = 16 (n = 17)^c^	Multi-Component n = 12 (n = 17)^c^
**Source of study**			
Journal article	24 (87.5)	14 (87.5)	10 (83.3)
Thesis	4 (14.3)	2 (12.5)	2 (16.7)
**Country of senior author**			
United States	23 (81.2)	13 (81.3)	10 (83.3)
Europe	4 (14.3)	2 (12.6)	2 (16.7)
Australia	1 (3.6)	1 (6.3)	-
**Level of evidence**			
Randomized controlled trial	10 (35.7)	3 (18.8)	7 (58.3)
Controlled trial	2 (7.1)	1 (6.3)	1 (8.3)
Historic control	1 (3.6)	-	1 (8.3)
Pre-post	9 (32.1)	7 (43.8)	2 (16.7)
Pre-post + single case randomization	1 (3.6)	-	1 (8.3)
Cross-sectional	4 (14.3)	4 (25.0)	-
Case series	1 (3.6)	1 (6.3)	-
**ISG format**			
Bulletin Board	9 (32.1)	4 (25.0)	5 (41.7)
Chatroom	5 (17.9)	5 (31.3)	-
Mailing list/newsgroup	2 (7.1)	1 (6.3)	1 (8.3)
Instant Messaging	2 (7.1)	1 (6.3)	1 (8.3)
Combination	6 (25.0)	3 (18.9)	3 (25.0)
Mailing list or bulletin board	2 (7.2)	2 (12.5)	-
Unclear	2 (7.2)	-	2 (16.6)
**ISG origin**			
Public, accessible	9 (32.1)	9 (56.3)	0 (0)
Closed, research ISG	17 (60.7)	7 (43.8)	10 (83.3)
Restricted access hospital	2 (7.1)	-	2 (16.7)
**Moderation status**			
Moderated	14 (50)	6 (37.5)	8 (66.7)
Some moderated	1 (3.6)	1 (56.3)	-
Not specified	13 (46.4)	9 (6.3)	4 (33.3)
**Type of moderation**	(n = 15)	(n = 7)	(n = 8)
Health professional	11 (73.3)	5 (71.4)	6 (75)
Don’t know	4 (26.7)	3 (28.6)	2 (25)
**Median duration intervention** (n = 29)^b^	16 wks (n = 23)	15.5 wks (n = 10)	17 wks (n = 13)
**Median longest follow-up** (n = 29)^b^ (from intervention commencement)	22 wks (n = 22)	26 wks (n = 12)	18.5 wks (n = 10)
			
**Condition** (n = 34)^c^			
Cancer	7 (20.6)	5 (29.4)	2 (11.8)
No disorder	7 (20.6)	7 (41.2)	-
Diabetes	2 (5.9)	1 (5.9)	1 (5.9)
Carers	4 (11.8)	-	4 (23.5)
Chronic illness	2 (5.9)	-	2 (11.8)
Neurological	2 (5.9)	-	2 (11.8)
Depression	2 (5.9)	2 (11.8)	-
Other mental disorder	2 (5.9)	1 (5.9)	1 (5.9)
Cardiovascular	1 (2.9)	1 (5.9)	1 (5.9)
Renal	1 (2.9)	1 (5.9)	-
HIV/AIDS	2 (5.9)	-	2 (11.8)
IVF	2 (5.9)	-	2 (11.8)
**Participant mean/median age** (n = 34)^c^			
11 to 17 yrs	3 (8.8)	2 (11.8)	1 (5.9)
18 to 25 yrs	4 (11.8)	3 (17.6)	1 (5.9)
26 to 40 yrs	5 (14.7)	2 (11.8)	3 (17.6)
41 to 65 yrs	11 (32.4)	4 (23.5)	7 (41.2)
Not certain	11 (32.4)	6 (35.3)	5 (29.4)
**Gender** (n = 34)^c^			
> 70% women	16 (47.1)	9 (56.3)	7 (46.7)
> 70% men	4 (11.8)	-	4 (25.0)
Neither gender > 70%	11 (32.4)	7 (43.8)	4 (50)
Don’t know	3 (8.8)	1 (6.3)	2 (11.8)
**Rural** (n = 34)^c^			
> 50% rural	1 (2.9)	0 (0)	1 (5.9)

^a^ Values are no. (%) unless otherwise specified.

^b^ Multiple samples receiving different intervention durations treated separately (one study: [24])

^c^ Multiple samples treated separately (six studies: [21,24-28])

#### Origin

The majority of studies were reported in published journal articles, and, in most cases, the senior author was located in the United States.

#### Interventions

The studies primarily employed bulletin boards, chatrooms, or mailing lists, either alone or in combination (see Table 3). Approximately two-thirds were closed ISGs, typically developed for research purposes. Half of the studies specified that the ISGs were moderated, and of these the majority of moderators were health professionals. The duration of the interventions ranged from 12 minutes to 12 months (median 16.5 weeks), and length of time to follow-up ranged from immediately post-intervention to 12 months post-intervention.

#### Participants

More samples were focused on ISGs for breast cancer than any other condition. In addition, a significant percentage of the samples related to depression and ISG use in those without a physical or psychological condition. As noted above, only two samples were exposed to depression ISGs. The median age of participants in the samples typically fell between 26 and 65 years. Some of the samples comprised college-aged or younger adolescents. None was concerned specifically with older people, although the median age of one sample was 64 years [28]. Significantly, only a minority of samples focused on men, whereas almost one half contained a predominance of, or all, women. Only one study focused on rural participants [47]; two others mentioned the inclusion of some rural residents [26,30].

#### Outcome Measures

Half of the studies (n = 14) used the Center for Epidemiologic Studies Depression Scale (CES-D) as an outcome measure, with the next most common measures (with two trials each) being the Symptom Checklist 90 (SCL-90) and the Beck Depression Inventory (BDI). Each of the remaining measures was administered in one trial only.

#### Study Quality

One third of the studies involved an RCT, and almost half of the 28 studies employed a control group. The majority of the remaining studies used a pre-post design. Of the 23 studies that used at least a pre-post design, only three (13%) used an ITT design, with a further study neither specifying if an intent-to-treat design was employed nor indicating the level of dropout if any [46]. Two of the four ITT studies [29,33] used the last observation carried forward method for treating missingness. The third inferred mood from initial and final posts on a bulletin board, thus ensuring that there was no dropout [32]. No study used multiple imputation for estimating missingness. Of the nine studies said to have employed an RCT design, only three [22,24,48] both adequately specified the randomization procedure and employed an appropriate method of randomization [51].

Intervention and control sample sizes ranged from 10 to 244 (median 46) and 30 to 236 (median 51), respectively, for samples derived from studies of at least pre-post test quality. Cross-sectional study sample sizes ranged from 158 to 2373 (median 230). Dropout among samples in studies of at least pre-post test quality ranged from 7.9% to 41.7% and 0% to 37% for intervention and control conditions, respectively. Of the 22 studies of at least pre-post design with some dropout, 46% (n = 10) compared the characteristics of completers and non-completers. All but one of these (n = 9, 90%) reported no difference in baseline characteristics for these groups.

### ISG Efficacy for Depression

The outcomes for single and multiple studies are discussed separately.

#### Single-Component Studies

Of the 17 intervention samples (16 studies) involving a peer-to-peer component alone or a cross-sectional design, 10 (59%) yielded a positive effect of the ISG on depressive symptoms. However, only two of these involved a controlled trial.

The largest number of single-component samples involved women with breast cancer (n = 5) [20,29-32]. Of these, four yielded significant effects of moderate to large size [20,29-31], and the fifth was associated with a small, significant association between board use and improved mood [32]. However, only one of these trials employed a controlled design [29].

Three samples (three studies) involved ISGs comprising members with a mental disorder, two of them depression [33-35]. One of these produced a positive result. In particular, Houston et al [34] found that more frequent depression ISG users were significantly more likely to recover from depression after adjustment for baseline depression severity and demographic variables. However, the study did not include a control group. The second depression ISG comparison involved the control arm of an RCT of an online cognitive behavior therapy intervention for depression in which a research bulletin board was used as a control condition [33]. There was no significant effect of the bulletin board.

There were two other single-component samples (2 studies) involving medical conditions, one of them involving a trial of an ISG for diabetes [36,37], the other the use of an ISG for renal patients undergoing dialysis [38]. The ISG did not produce an effect on depressive symptoms in either of these studies, but the latter involved only three cases.

Finally, seven samples (six studies) involved people with no psychological or physical disorder [27,39-43]. Three samples (two studies) involved experimental studies of the effect on mood of online communication between peer dyads [27,39]. Two of these reported a positive effect of the dyad on mood. The remaining four samples (four studies) involved cross-sectional studies of survey data designed to investigate the association between frequency of chatroom use and mood in community samples. Two of these studies involved university communities and found that higher chatroom use predicted lower depression [40,43]. A third, cross-sectional study of general users on the Internet did not find an association between frequency of use and mood but employed a dichotomized measure of frequency and may therefore have lacked statistical power [42]. The final study, which involved adolescents aged 11 to 16 years, found a reverse effect, with higher Internet use being associated with a higher level of depressive symptoms [41]. In summary, there is weak evidence that chatroom use among people without a disorder may be associated with lower levels of depression, but the quality of evidence is poor and the findings inconsistent.

#### Multi-Component Studies

Of the 17 samples (12 studies) that involved intervention components in addition to the ISG, only two (12%) reported a positive effect [21,28]. The first, involving a homogenous group of patients with Parkinson’s disease, employed a pre-post design only and incorporated a health professional education component as well as the ISG [28]. The second, involving heart recipients, employed a historical control differing in depression severity and comprised many potentially active components in addition to the ISG, including stress skills training [21].

#### Association Between Positive Results and Study Characteristics

Multi-component studies were significantly less likely to yield significant, positive outcomes than stand-alone interventions and cross-sectional studies (Fisher exact test, *P* = .01). Breast cancer ISGs were more successful than other ISGs (Fisher exact test, *P* = .02), but most of the breast cancer studies originated from a single research group. Outcome was not affected by the use of synchronous (chatroom) compared to asynchronous (bulletin board, listserv/newsgroups) ISGs (Fisher exact test, *P* = .99), whether or not the study reported using a moderator (Fisher exact test, *P* = .72) or whether the board was public, research, and/or restricted access (Fisher exact test, *P* = .11). There was no effect on outcome for the duration of the intervention (Mann-Whitney U = 57, *P* = .23) or the length of follow-up (Mann-Whitney U = 75.5, *P* = .83). Nor was there a significant association between age (25 years and younger vs older) and success, but there were few studies of young people (Fisher exact test, *P* = .64). Considering only the samples that were predominantly comprised of males (n = 4) or females (n = 16), there was no association between outcome and sex (*P* = .59), but the sample size of males was very small.

With respect to study quality, there was a trend toward an association between lower design quality and positive outcomes, with 19% (n = 3) of samples involving controlled comparisons (RCT, controlled trial, historic control) and 53% (n = 9) of uncontrolled effects yielding significant positive findings. However, this association fell short of statistical significance (Fisher exact test, *P* = .07). A similar non-significant trend (Fisher exact test, *P* = .13) was noted for samples involving RCTs compared to other designs. In the latter case, only 17% (n = 2) of the RCTs yielded a positive effect and none of these employed an ITT design. By contrast, 48% (n = 10) of the lower-quality trials yielded significant positive outcomes. There was no association between total sample size of study intervention groups and outcome (Mann-Whitney U = 62, *P* = .26).

## Discussion

The most salient finding of this review was the paucity of high-quality studies of the impact of depression or other ISGs on depression outcomes. Only a minority of the identified studies employed a control group, and two-thirds of RCTs either failed to use an adequate method of randomization or failed to specify the method of randomization. In addition, only 13% of studies of at least pre-post quality used an ITT analysis, and no study used multiple imputation for treating missingness. This low level of quality is a cause for concern, particularly given the trend toward an association between significant positive findings and low design quality.

Despite the apparent popularity of the Internet as a source of support for people with depression, there were only two studies of the effectiveness or efficacy of depression ISGs in improving mood. One comprised the control arm in a study of the effectiveness of a psychological therapy, and the other involved an uncontrolled multi-time-point study of an existing public depression ISG. Although the findings from the latter study were promising, neither study was of sufficient quality to evaluate whether depression ISGs improve or do not improve depression outcomes. Clearly, there is a need to undertake an RCT of the effect of a depression ISG on depression status.

Although there were more studies of the effect on depression for ISGs for conditions other than depression, many of these studies were of low quality and almost 50% employed multi-component interventions of which the ISG was only one component. Indeed, only two studies employed both a controlled design and a single-component intervention [27,29]. The first involved a structured 12-week breast cancer newsgroup intervention facilitated by a psychologist. There was a greater reduction in depressive symptoms among the ISG than the control group using ITT analyses. The second involved a sample of well adolescents and a sample of well college students who, after exposure to a negative mood induction manipulation, were provided with the opportunity to interact online with an unknown peer. There was an improvement in mood for the adolescents assigned to online peer interaction relative to control adolescents, but no such effect for college students. Thus, the results of the two highest quality studies are encouraging and suggest that further studies of ISGs of all types are warranted.

The finding that breast cancer ISGs were significantly more likely to be associated with positive results than ISGs of other types requires further investigation given that women with breast cancer are known to be at increased risk of depression [52]. If found to be effective in reducing depressive symptoms, such ISGs could provide an important mental health self-care and prevention tool for women with breast cancer. However, the status of the current results is unclear given that the majority of findings were derived from one research group and the studies were typically of low quality.

The finding that chatroom use tends to be associated with lower levels of depression among participants without depression or other medical conditions raises the possibility that chatroom usage may protect against depression in universal samples of members of the community. However, much of the evidence is based on cross-sectional surveys. Thus, the direction of causation cannot be determined, and chatroom usage may be associated with other behaviors and these rather than the chatroom use may mediate the depression levels.

Theoretically, online support groups could be particularly relevant and appropriate for users who are isolated or not able to access conventional or face-to-face services, either due to lack of mobility or geographic location. It is therefore of some concern that none of the studies investigated ISGs among older people and that only one study specifically focused on the effectiveness of an ISG for rural participants.

### Limitations

A limitation of this study is that it does not include trials published after July 2007. To investigate this, a further search was conducted by the first author incorporating the time period from August 2007 to May 2009 and using the same search terms employed in the reported searches but limiting results to those incorporating the terms “depression” or “depressive” or “mood.”

After excluding a published study reporting data from a dissertation already incorporated into the review [53], 14 new relevant papers were identified. Of these, six involved experimental studies [54-59] and the remainder were non-experimental [60-67]. No new descriptive studies of depression ISGs were identified. Of the experimental studies, all but two [54,55] incorporated potentially active components in addition to an ISG. Only one of the six employed an ITT design [58], and although three were RCTs [56-58], none specified the method of randomization. The remaining three experimental studies were controlled trials [54,55,59], but one employed a non-contemporaneous control [54]. Of the two single-component studies, one involved an ISG for Spanish-speaking immigrant women with breast cancer [54] and the other an ISG for Asian American women with a lesbian or bisexual orientation [55]. Neither resulted in a positive effect on depressive symptoms relative to a control.

Of the four multi-component trials [56-59], three reported a greater reduction in depressive symptoms in the intervention group [57-59]. The first of these studies involved an ISG and educational films for people with chronic pain or burnout ([57], RCT), but the effect was not sustained at follow-up. The second employed a discussion group in addition to a therapist-facilitated online group and an offline cognitive behavioral therapy program, but the latter is a known effective treatment for depression ([58], RCT). The third comprised a computer and Internet educational program for older people that incorporated, but was not limited to, participation in forums and virtual communities ([59], controlled trial). The remaining multi-component trial found no effect of a complex intervention incorporating an ISG component for rural-residing women with a chronic illness ([56], RCT). This study found that an intensive intervention involving peer-to-peer online support, expert-facilitated online group discussion, and online expert advice resulted in no greater reduction in depression than an information intervention alone or no intervention [56]. The 11 non-experimental studies identified investigated the relationship between chatroom (unspecified) use and depression, and most used a cross-sectional design. The findings were mixed. In summary, studies published since mid-2007 shed little additional light on the effectiveness of ISGs in reducing depressive symptoms and provide no further evidence concerning the efficacy of depression ISGs.

### Conclusions

There is a need for high-quality research to investigate the effect of ISGs on depression outcomes. We acknowledge that there are significant challenges associated with designing and undertaking efficacy studies of ISGs. We acknowledge too that the appropriateness and feasibility of conducting such research on online self-help groups have been questioned [68]. However, we believe that creative researchers, together with consumers, can find a way to shed further light on an issue of unquestionable practical significance for millions of consumers worldwide.

## Abbreviations

ISG: Internet support group
ITT: intent to treat
RCT: randomized controlled trial
